# A patient journey audit tool (PJAT) to assess quality indicators in a nuclear medicine service

**DOI:** 10.1007/s00259-024-06627-8

**Published:** 2024-02-10

**Authors:** Kunthi Pathmaraj, Jessica Welch, Wesley Ng, Danny Lee, Sze Ting Lee, Anita Brink, Maurizio Dondi, Diana Paez, Andrew M. Scott

**Affiliations:** 1 Department of Molecular Imaging and Therapy, Austin Health, Level 1, Harold Stokes Building, Studley Road, Melbourne, Victoria 3084 Australia; 2 Olivia Newton-John Cancer Research Institute, Melbourne, Victoria Australia; 3 School of Cancer Medicine, La Trobe University, Melbourne, Victoria Australia; 4 School of Health and Biomedical Science, RMIT University, Melbourne, VIC Australia; 5 Department of Medicine, University of Melbourne, Melbourne, Australia; 6 Division of Human Health, International Atomic Energy Agency, Vienna, Austria

**Keywords:** Patient journey audit tool, Quality indicators, Clinical governance, Nuclear medicine, PET

## Abstract

**Purpose:**

To develop a nuclear medicine specific patient journey audit tool (PJAT) to survey and audit patient journeys in a nuclear medicine department such as staff interaction with patients, equipment, quality of imaging and laboratory procedures, patient protection, infection control and radiation safety, with a view to optimising patient care and providing a high-quality nuclear medicine service.

**Methods:**

The PJAT was developed specifically for use in nuclear medicine practices. Thirty-two questions were formulated in the PJAT to test the department’s compliance to the Australian National Safety and Quality Health Service Standards, namely clinical governance, partnering with consumers, preventing and controlling health care infection, medication safety, comprehensive care, communicating for safety, blood management and recognising and responding to acute deterioration. The PJAT was also designed to test our department’s adherence to diagnostic reference levels (DRL). A total of 60 patient journey audits were completed for patients presenting for nuclear medicine, positron emission tomography and bone mineral density procedures during a consecutive 4-week period to audit the range of procedures performed. A further 120 audits were captured for common procedures in nuclear medicine and positron emission tomography during the same period. Thus, a total of 180 audits were completed. A subset of 12 patients who presented for blood labelling procedures were audited to solely assess the blood management standard.

**Results:**

The audits demonstrated over 85% compliance for the Australian national health standards. One hundred percent compliance was noted for critical aspects such as correct patient identification for the correct procedure prior to radiopharmaceutical administration, adherence to prescribed dose limits and distribution of the report within 24 h of completion of the imaging procedure.

**Conclusion:**

This PJAT can be applied in nuclear medicine departments to enhance quality programmes and patient care. Austin Health has collaborated with the IAEA to formulate the IAEA PJAT, which is now available globally for nuclear medicine departments to survey patient journeys.

## Introduction

Nuclear medicine services are being increasingly sought for both diagnostic imaging procedures and radionuclide therapy in the evaluation of oncological, cardiac, neurological and endocrinological conditions and infections. The patient journey in a nuclear medicine service comprises of booking the procedure, preparation for the procedure prior to the appointment, arrival and registration on the day of the appointment, patient interview for clinical history and undergoing the procedure itself which includes radiopharmaceutical administration, scanning, report generation and dispatching of results.

At each of these points in the patient journey, quality indicators should be exerted to ensure patient safety is a critical focus, procedures are conducted according to appropriate protocols and the clinical question is addressed in the final report. Quality standards in health care are increasing; therefore, knowledge and experience in the use of tools for quality management must grow accordingly. A nuclear medicine service should have an established quality and safety programme that aligns to the quality principles of its organisation, as well as the national and international health standards/guidelines.

The International Atomic Energy Agency (IAEA) has the most publications in this area, with their flagship quality programme, QUANUM (Quality Management Audits in Nuclear Medicine Practices), developed to audit a nuclear medicine department as a whole and is based on international guidelines from Society of Nuclear Medicine and Molecular Imaging (SNMMI), European Association of Nuclear Medicine (EANM) as well as prior publications from IAEA [1]. QUANUM contains relevant checklists to audit all aspects of nuclear medicine practices including clinical practice, management, operations and services.

The QUANUM programme provides guidelines and framework for maintaining a strong quality programme in a nuclear medicine service. QUANUM has sought to instil the culture of quality by encouraging the conduction of annual systematic audits, the adoption of a culture of regular analyses and reviews of internal processes and the introduction of a quality audit process that is patient-oriented, systematic and outcome-based. Adopting a culture of auditing through peer review is essential and enhances the contribution of nuclear medicine to safe practice and optimal patient care. The IAEA publications analysing the QUANUM audit missions show that QUANUM audits have overall contributed to significant improvements of clinical practice and services to patients around the world [2, 3].

A review of Austin Health processes showed that the hospital uses a clinical audit portal to conduct patient journey audits in clinical areas, namely in the wards, intensive care unit and emergency department. These audit tools were not fit for the purpose of auditing patient journeys and clinical service delivery in a nuclear medicine department.

Recognising the need for ongoing quality management systems, internal audits and the implementation of the PDCA (plan, do, check and act) cycle in a nuclear medicine service to optimise patient care and minimise risks, the Department of Molecular Imaging and Therapy at Austin Health developed an in-house, nuclear medicine specific, patient journey audit tool (PJAT) that could be used to audit patient journeys in a nuclear medicine service. The PJAT was developed by drawing on the IAEA QUANUM principles and the clinical audit tools utilised by Austin Health and customised specifically for use in nuclear medicine practices. In developing the PJAT, the focus was kept on developing quality audit processes in nuclear medicine that can assist nuclear medicine departments/laboratories in maintaining or improving the quality of service for their patients, and thus lending itself to use by nuclear medicine services globally.

The main scope for the PJAT is to be able to review and evaluate the quality of all elements involved in a patient journey, including staff interaction, equipment and procedures, patient protection and safety, as well as its interaction with external service providers.

## Materials and methods

The PJAT questions were formulated to test the department’s compliance to the Australian National Safety and Quality Health Service Standards (NSQHS) (Table 1), professional guidelines (e.g. diagnostic reference levels, DRL) mandated by regulatory bodies such as the Australian Radiation Protection and Nuclear Safety Agency (ARPANSA) and guidelines set by the Patient Safety and Clinical Excellence Unit of Austin Health. The PJAT was also designed to audit clinical and laboratory procedures, COVID-19–related governance and radiation safety practices. An essential feature of the PJAT audit tool is its ability to be used as part of the PDCA cycle. The audit tool was designed to have the ability to clearly identify gaps and areas that do not quite comply against audited parameters and therefore target these areas for improvements.

**Table 1:** Australian National Safety and Quality Health Service (NSQHS) Standards [4]

Standard	Description
1	Clinical governance, which describes the clinical governance and safety and quality systems that are required to maintain and improve the reliability, safety and quality of health care and improve health outcomes for patients.
2	Partnering with consumers, which describes the systems and strategies to create a person-centred health system by including patients in shared decision-making, to ensure that patients are partners in their own care and that consumers are involved in the development and design of quality health care.
3	Preventing and controlling infections, which describes the systems and strategies to prevent infection, manage infections effectively when they occur, limit the development of antimicrobial resistance through prudent use of antimicrobials (as part of effective antimicrobial stewardship) and promote appropriate and sustainable use of infection prevention and control resources.
4	Medication safety, which describes the systems and strategies to ensure that clinicians safely prescribe, dispense and administer appropriate medicines to informed patients and monitor use of the medicines.
5	Comprehensive care, which describes the integrated screening, assessment and risk identification processes for developing an individualised care plan, to prevent and minimise the risks of harm in identified areas.
6	Communicating for safety, which describes the systems and strategies for effective communication between patients, carers and families, multidisciplinary teams and clinicians and across the health service organisation.
7	Blood management, which describes the systems and strategies for the safe, appropriate, efficient and effective care of patients’ own blood, as well as other supplies of blood and blood products.
8	Recognising and responding to acute deterioration, which describes the systems and processes to respond effectively to patients when their physical, mental or cognitive condition deteriorates.

The PJAT was a hospital department quality evaluation project, and there was no requirement for formal Hospital Ethics Committee approval of the project.

Table 2 illustrates the 32 questions that were formulated in the PJAT database, including the method of extracting the responses to each of the questions. Two sets of random patient journey audits were conducted during a consecutive 4-week period. Initially, 60 patient journey audits were conducted, 20 patients in nuclear medicine (NM), 20 patients in positron emission tomography (PET) and 20 patients in bone mineral density (BMD), to sample the wide range of procedures performed in the department. A further subset of 120 patient journey audits was completed for common NM and PET procedures to critically assess key aspects of the patient journey in the department. This subset constituted of 20 bone scans, 20 myocardial perfusion imaging studies, 20 lung VQ scans, 40 whole-body PET scans and 20 FDG brain PET scans. In addition, 12 laboratory procedures that required withdrawal and re-administration of patient’s blood (2 gastrointestinal bleed studies and 10 cardiac gated blood pool scans) were reviewed to test compliance of the Blood Management Standard (NSQHS Standard 7). The questions in the PJAT were addressed at different time points of a patient journey in the department and were asked of the patient by the receptionist, nuclear medicine technologist and nursing staff. One technologist was responsible for each individual patient journey audit and ensured that all the data that was collected by other staff was entered accurately in the PJAT database.

**Table 2:** PJAT questions

No	Question
1	What was the time from request received to being triaged to senior technologists? (i)
2	What was the time taken for senior technologists to protocol the request? (i)
3	What was the time taken from receiving the protocoled request to booking (patient notified)? (i)
4	Were 3 patient identifiers used to identify the patient correctly at reception upon arrival? (ii)
5	Did the patient receive appointment and preparation information in a format that was easily accessible and easy to understand? (ii)
6	Did the patient adhere to preparation instructions? (ii)
7	Was verbal informed consent obtained as appropriate? (ii)
8	Was written informed consent obtained as appropriate? (ii)
9	Were 5 moments of hand hygiene practiced when dealing with the patient? (iii)
10	Was fall risk assessment performed? (iv)
11	Was the patient assessed as risk of fall? (iv)
12	For patients at risk of fall, was appropriate care taken to prevent a fall? (iii)
13	For inpatients, was there a completed interdepartmental transfer form or appropriate handover? (v)
14	For those inpatients who require a nurse escort, was there adequate handover to MIT nursing staff? (vi)
15	Were allergies noted? (iv)
16	Was sedation administered according to protocol? (vii)
17	If applicable, were pharmaceuticals administered according to protocol? (vii)
18	Were 3 patient identifiers used to identify the patient correctly before the radiopharmaceutical administration? (iv)
19	Was the outpatient injection area/uptake room clean? (iv)
20	Was the scanner room cleaned prior to the patient entering? (iv)
21	Were blood collection vials labelled correctly with patient name, URN and date? (iii)
22	Was the radiopharmaceutical syringe and dose slip labelled and dated correctly? (iv)
23	For radiopharmaceutical administration, were the 7 rights of medication administration adhered to? (Right patient, right drug, right dose, right route, right time, right documentation, right reason) (iv)
24	Was the radiopharmaceutical administered activity within prescribed diagnostic reference level (DRL) specifications? (iv)
25	When applicable, were patient observations performed and recorded? (iii)
26	Was relevant personal protective equipment (PPE) used at time of procedure? (iii)
27	Was the department aware of the next review date of patient? (iv)
28	Was the intravenous (IV) cannula removed when the outpatient left the department? (iii)
29	Did the patient receive relevant precautions when leaving the department? (iv)
30	Was a radiation alert sticker placed on inpatient history for ward patients and/or was radiation alerts recorded in the hospital’s electronic medical record? (iv) (viii)
31	For inpatients, was there adequate handover to ward staff of any clinical interventions that took place whilst in MIT? (vi)
32	What was the time taken from scan completion to report generation? (i)
	Key: i. Parameter extracted from department information system ii. Information obtained directly from the patient by the technologist iii. Self-assessment by technologist iv. Parameter extracted from clinical and technical datasheet v. Information obtained from the inpatient notes by the technologist vi. Confirmed by the nursing staff vii. Technologist observed the nurse administer the medication viii. Information extracted from the hospital’s electronic medical record

All the 32 questions in the PJAT were applied to the first 60 patient journey audits and the subsequent 120 audits that were conducted for the subset of common procedures in NM and PET.

The Australian Clinical Governance Standard recognises the importance of governance, leadership, culture, patient safety systems, clinical performance and the patient care environment in delivering high-quality care. For purposes of the patient journey audit, this standard was measured by reviewing the compliance to the department’s clinical governance framework including following standard operating procedures (SOP) for patient identification, radiopharmaceutical administration, scanning, scan reporting, hand hygiene measures to reduce risk of health care-associated infections, prescribed DRL, radiation safety measures for staff and patients, correct procedures for personal protective equipment and reducing falls risk.

The standard protocol for a procedure in our department (including NM procedure, PET scan or BMD scan) is for the technologist to complete the clinical and technical datasheet (CTDS) by interviewing the patient and recording the clinical history including various other aspects of the patient journey. These aspects included patient identification, informed consent, risk assessment, review date, allergies, clean environment checks, imaging times and the administered dose of the radiopharmaceutical. During the patient interview, the assigned technologist asked the patient if (i) the patient had received and followed the preparation for the scan (questions 5 and 6) and (ii) the three patient identifiers were checked by the reception staff on arrival (question 4).

Radiation precautions following a diagnostic procedure are usually communicated verbally; however, if an outpatient is attending another medical appointment on the same day following the scan, a radiation precaution document is provided to the patient to take to their next appointment. For inpatients, in addition to providing verbal instructions, the radiation precautions are also recorded in the patient’s electronic medical record (EMR) to ensure adequate communication with ward staff. The provision of the radiation precaution document is also recorded on the clinical and technical datasheet which is scanned and electronically stored on the radiology information system (RIS) for retrospective analysis (questions 7, 8, 10, 11, 15, 18, 19, 20, 24, 27 and 30).

Auditing the infection control measures during the PJAT required the technologist to identify any missed infection control measures such as the correct application of personal protective equipment (PPE) and observation of the five moments of hand hygiene [5] (questions 9 and 26). Patient recliner chairs in the administration areas, scanner beds and imaging equipment were cleaned between each patient use and recorded on the clinical and technical datasheets which were used to audit compliance of the technologist for questions 19 and 20.

PJAT questions 7, 8, 18, 22 and 23 apply to medication safety and hence were used to audit governance processes around the administration of radiopharmaceuticals. The administered dose of the radiopharmaceutical was calculated by decay correcting the pre- and post-injection syringe to the time of injection with the use of an in-house, web-based dose calculator. The exact administered dose was then compared against the DRL specifications (question 24). To assess the compliance of the medication safety SOPs and guidelines when administering sedatives, the auditing technologist observed the administration of the sedative by nursing staff to ensure the medication safety standards have been met (questions 15, 16 and 17). More information was gathered from the patient electronic medical record or scanned medical record for auditing purposes (questions 13, 14 and 31).

The time taken from the receipt of the patient’s request to be triaged and booked for a procedurewas monitored to ensure requests were booked in a timely fashion (questions 1, 2 and 3). The patient journey time in the department was retrospectively calculated based on the patient arrival time and the time at which their procedure was completed. Additionally, the duration between scan completion and report generation was also obtained from the department information system (question 32). Reports are sent out electronically by a secure message delivery network (SMDN), allowing the secure communication of health information from one health care provider organisation to another, as soon as the report is finalised. For those referrers without access to SMDN, reports are either sent by encrypted email or posted. The patient journey audit reviewed the results dissemination process including the communication of significant and unexpected findings.

Another important aspect of the patient journey survey was to assess patient satisfaction of the nuclear medicine service. Our department utilises a booking software application which has the ability to send the appointment date and time, procedure information, patient preparation, COVID-19 questionnaire and the ability for the patient to provide feedback electronically. The booking software application automatically sends a short message service (SMS) to the patient once their scan is completed providing the patient with the option to participate in a patient satisfaction survey. During the 4-week audit period, 108 patients provided feedback. Note that feedback was not necessarily provided by the 180 patients who were randomly audited by the PJAT. Key questions in the survey include the following:

1. Did the staff introduce themselves?
2. Did the staff explain the procedure to you in a clear fashion?
3. Were you treated with courtesy and respect?
4. Did the staff give you an opportunity to ask questions before the procedure?
5. Were you satisfied with the service?
6. What was your experience with the digital app?

## Results

The data collated from the patient journey audits was categorised according to the NSQHS standards, graphed and reviewed.

### Standard 1—clinical governance

**Fig. 1:**
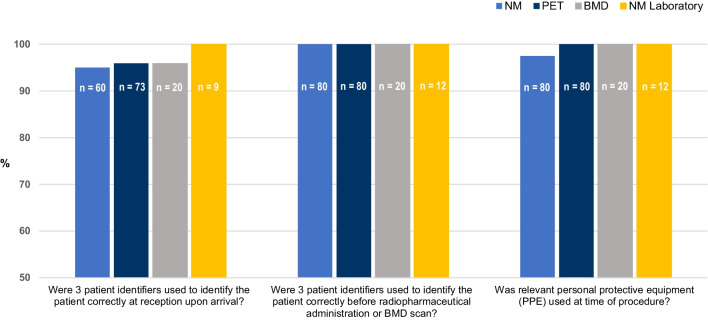
Standard 1 clinical governance. Ensuring safety and quality systems are followed to maintain reliability, safety and quality of health care. The *n* value is smaller for the first set of data because inpatients do not report to the department reception, they report directly to the nursing station

Very high compliance was noted for the 180 patient journey audits for adherence to departmental SOP through all aspects of the patient journeys. In two instances, the sterile intravenous (IV) cannulation procedure was not followed according to the SOP, as eye protection was not worn (Fig. 1). High level of compliance of greater than 95% was noted for recording three patient identifiers by reception staff, and 100% compliance was noted for identifying the patient correctly prior to the procedure and radiation exposure.

### Standard 2—partnering with consumers

**Fig. 2:**
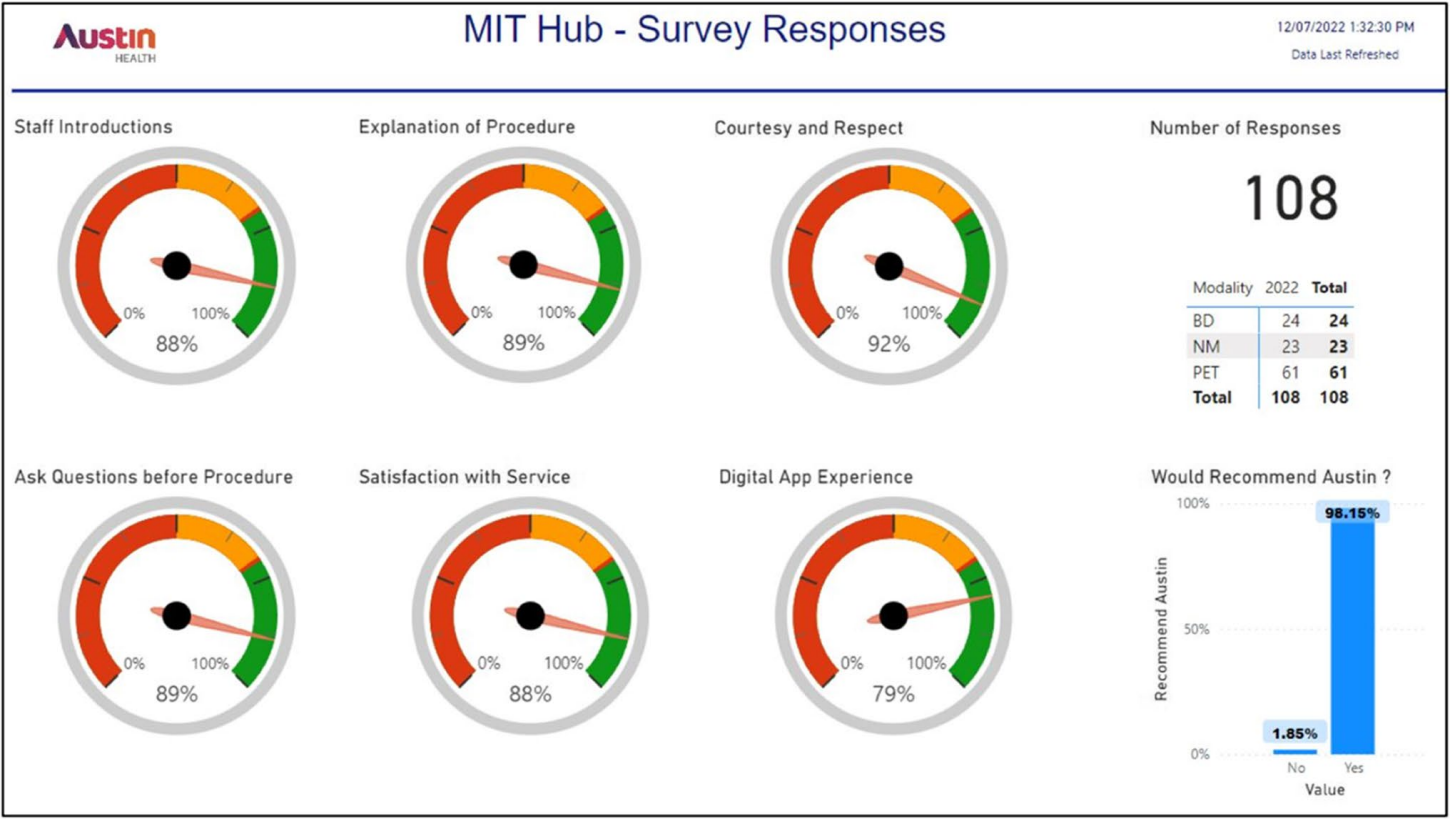
Standard 2 partnering with consumers. Survey responses from patients who attended the department during the audit period

For the audit period, more than an 85% satisfaction rate was noted for staff introduction, explanation of procedure, courtesy and respect, asking questions before procedure and overall satisfaction with the service (Fig. 2). The digital application experience was recorded at just under 80% satisfaction.

### Standard 3—preventing and controlling health care-associated infections

During the audit period, attention was paid closely to audit the compliance of staff with measures that are expected to be taken to prevent and control health care-associated infections. Results demonstrated high compliance for all the parameters that were tested, namely (i) was the iv cannula was removed when outpatients left the department, (ii) was relevant PPE used at the time of the procedure, (iii) was the scanner room cleaned before patient use, (iv) was the out-patient injection area and uptake rooms clean when patients entered and (v) were five moments of hand hygiene practiced when dealing with the patient. It was found that the department was complying with hospital infection control policies and exerting due diligence to minimise the risk of infection and COVID-19 transmission.

### Standard 4—medication safety

**Fig. 3:**
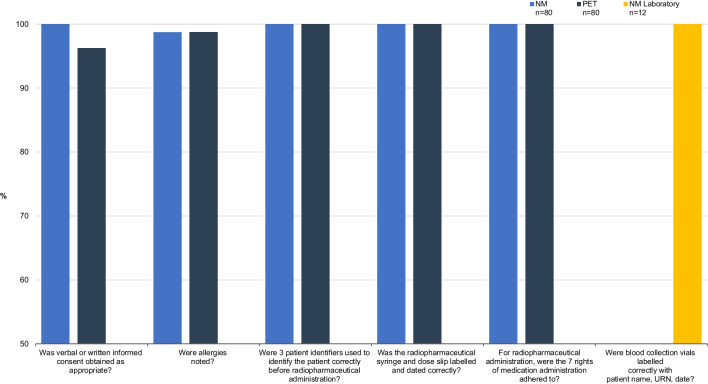
Standard 4 medication safety. High compliance was observed for all aspects of medication safety in NM and PET. BMD was not included since no medication/radiopharmaceutical is administered for DXA scans

High compliance was noted for all assessed aspects of medication safety (Fig. 3).

Our department conducts annual assessments of DRLs for all diagnostic procedures. The facility reference levels (FRL) are reviewed against DRL recommended by ARPANSA, with a view to ensure administered radioactivity is within the prescribed DRL whilst maintaining optimal image quality. Administered radioactivity was reviewed for all patients in the PJAT and for an in-depth analysis, a subset of 120 common procedures in NM and PET procedures were extracted and compared against the DRL guidelines set by ARPANSA.

**Fig. 4:**
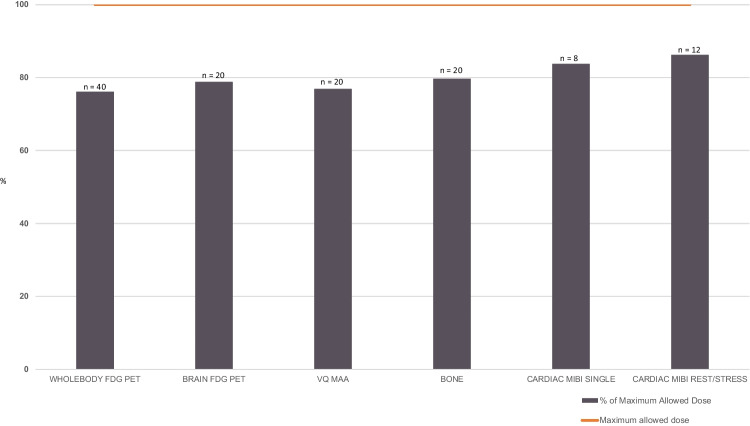
Patient safety. Diagnostic reference levels for administered radiopharmaceutical dose. The chart demonstrates the average administered dose for each of the common procedures in the subset of 120 patients, where 100% depicts the maximum allowed dose as stipulated by ARPANSA

Our department demonstrated excellent DRL compliance to the ARPANSA guidelines for all the procedures during the audit period, and the subset of 120 common NM and PET procedures demonstrated that we are below the maximum allowed dose (Fig. 4). The average dose length product (DLP) for studies requiring a low dose CT for attenuation correction and anatomical correlation was also well within the maximum dose allowed by the ARPANSA guidelines.

### Standard 5—comprehensive care standard

In NM, PET and BMD, for patients who were assessed as falls risk, appropriate care was taken to prevent/minimise the risk of fall.

### Standard 6—communicating for safety

Patient identification by three-point identifier checks, as well as procedure matching, revealed high compliance rates, assuring that the right patient for the right procedure was receiving the radiopharmaceutical injection. In one instance in BMD, there was no record in RIS that the patient was identified by administration staff at the time of registration. There was 100% compliance for identifying the patient by three-point identification prior to radiopharmaceutical administration for NM and PET procedures and scanning the patients for BMD. High compliance was noted for all the other parameters that were assessed for the communication for safety standard. For myocardial stress test patients, compliance was high for all aspects of work practice including three identifiers being used to check patient identity prior to radiopharmaceutical administration. To assist with communication of results, especially significant and unexpected findings, patients are asked if they are aware of their next review date with their specialist, general practitioner or outpatient clinics. It was demonstrated that 72% of PET patients and 37% of NM patients were aware of their review appointment. The remaining patients were unsure about their follow-up appointment.

### Standard 7—blood management standard

During the audit period, 12 procedures (2 gastrointestinal studies and 10 cardiac gated blood pool studies) required venous blood being collected from the patient via an intravenous cannula, radiolabelled and readministered to the patient. In all 12 instances, blood collection vials were labelled correctly with the patient name, unit record number and date of the procedure. Additionally, patient identification was performed twice for each of these procedures, once prior to withdrawal of blood and once prior to re-injection of radiolabelled blood.

### Standard 8—recognising and responding to acute deterioration

There were no patients in the audit period that fell into this category.

#### Patient journey time in the department and time taken to generate a report

**Fig. 5:**
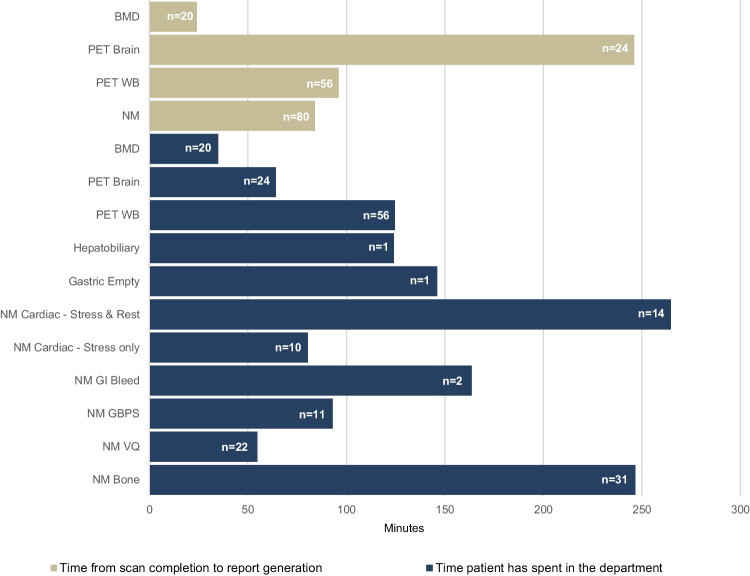
The time spent by patients in the department from arrival to completion of the study and time taken from scan completion to report generation

In addition to evaluating the department’s performance against the Australian NSQHS, the patient journey audit also enabled a review of the duration of the time spent by patients in the department for a range of procedures (arrival time to completion of the procedure) and the time taken for a report to be issued after completion of a scan. This analysis was conducted for a total of 180 patient journey audits, as well as the subset of 12 patient journey audits that were conducted to audit the blood management standard. The average time a patient spends for a particular procedure is shown in Fig. 5. Report turnaround times were very efficient, and the mean time from scan completion to report generation was 1.4 h for NM reports, 1.6 h for PET WB reports and 4.1 h for PET brain reports. These timeframes refer to reports being accessible on PACS for internal distribution within Austin Health and being distributed by the SMDN for externally referred patients.

## Discussion

The Austin Health PJAT was used successfully to audit 180 patient journey audits and demonstrated a high compliance to the national health standards and adherence to DRL stipulations. The patient journey audits confirmed that the department is delivering a high-quality nuclear medicine service. The PJAT provides an efficient approach to establishing compliance and patient-focused quality parameters in a busy nuclear medicine department.

Self-assessment and data-driven decision-making is a critical aspect of a quality programme in any nuclear medicine service which can enhance the quality of the service and ensure alignment with local, national and international standards for best nuclear medicine practice and patient-centred care. The European Commission has published the results of the QuADRANT study (Quality Improvement Through Clinical Audit in Diagnostic Radiology, Radiotherapy and Nuclear Medicine) illustrating a snapshot of the current situation of clinical auditing in EU countries [6]. The publication suggests that regular clinical audits help close the gap between everyday clinical practice as well as describe what is recommended in the current literature. Delgado et al. [7] shine light on the necessity of a clinical audit to improve the nuclear medicine service in many nations and summarise recent findings of the QuADRANT study. National co-ordination, regulatory control, involvement of a national professional body, consideration of the barriers and enablers, accreditation and certification and education of health professionals have been found to be the key aspects of successfully implementing a clinical audit.

Berman et al. [8] have published the outcomes of a survey to assess the patient experience before and after introducing an in-house radioiodine therapy clinic allowing for a direct consultation between the nuclear medicine specialist and the patient. The patient journey through the clinic and subsequent radioiodine treatment was surveyed pre and post-treatment with favourable outcomes post-treatment relating to patient satisfaction ratings. Similar results were achieved by Moncayo et al. [9]. However, these studies are limited to the radioiodine therapy procedure and are unable to provide a wider perspective or overview of the patient journeys for other services provided by a nuclear medicine department.

These publications establish the importance of a clinical audit and that it is well recognised internationally and emphasised by many professional bodies. There is a lack of published guidelines to comprehensively audit the patient’s experience through nuclear medicine services where diagnostic and radionuclide therapy procedures are performed on a regular basis.

The PJAT we have developed was demonstrated to be an efficient audit tool to conduct patient journey audits and was developed based on the IAEA QUANUM principles to specifically assess if a nuclear medicine service in Australia meets the requirements of the Australian NSQHS standards. The PJAT is able to assess quality indicators in administrative processes such as booking of the appointment, patient preparation, correct patient identification and procedure matching, patient interview prior to a procedure, appropriate management of human resources in regard to training and clinical competence, governance around infection control measure, nuclear medicine specific aspects such as radiation safety, radiopharmaceutical administration and adherence to prescribed DRL as well as assess the time taken from the receipt of the imaging request to performing the scan and generating the scan report. The results of the patient journey audits can also be used as part of the PDCA cycle to identify gaps and hence encourage continuous improvement of all aspects of a nuclear medicine service.

Whilst the Austin Health PJAT was developed initially to test compliance of nuclear medicine practices against Australian health standards and DRL guidelines, it can easily be adapted by any nuclear medicine service for their own needs, to review their practices and ensure compliance with either local regulations or international guidelines set by organisations such as the IAEA. Towards this end, Austin Health and IAEA have collaborated to formulate a patient journey audit tool (IAEA PJAT) that draws on the fundamental principles of the Austin PJAT but is modified to accommodate nuclear medicine departments globally. The IAEA convened a group of experts in quality management to develop the IAEA PJAT. Questions in the IAEA PJAT were formulated to audit processes around all aspects within a nuclear medicine service including the assessment of the request/referral and justification of the procedure, triaging and booking the appointment, providing patient instructions and appointment details, assessing the clinical history, obtaining patient consent and preparing for procedures. Further, questions were developed to monitor radiopharmaceutical administration, the imaging, laboratory or treatment procedure; the management of adverse or unexpected events and deviations from standard practice. Finally, questions were built in the audit tool to monitor post-procedure instructions for both diagnostic and therapeutic procedures, including patient release and or discharge, report generation and result dissemination.

The IAEA PJAT has now been added to the QUANUM portfolio of products on the Human Health Campus website of the IAEA [10]. The IAEA-PJAT provides an opportunity for nuclear medicine departments globally to audit all the phases of the patient journey through a nuclear medicine procedure and nuclear medicine departments have the flexibility to tailor the questions in the audit tool and adapt it to align with their specific service requirements.

## Conclusions

Our department has successfully developed a patient journey audit tool (PJAT) that can be easily implemented as part of a nuclear medicine quality programme. The results of the 180 patient journey audits conducted show that our department is complying with the expectations set by government quality standards and guidelines set by professional nuclear medicine societies and national regulatory bodies. The IAEA PJAT has now been developed and made accessible on the IAEA website which allows global nuclear medicine services to use this patient journey audit tool for use in their nuclear medicine practices, identify gaps in processes and strive for excellence in quality to ensure optimal patient care.

## Data Availability

All data supporting the findings of this study are available within the paper.

## References

[1] Arends AJ, Baigorria SA, De Castro R, Dondi M, Estrada Lobato E, Giammarile F, Marengo M, Paez D, Pathmaraj K, Solanki K, Torres Aroches LA, Warwick JM. IAEA Human Health Series ISSN 2075-3772; No. 33. QUANUM 3.0: An updated Tool for Nuclear Medicine Audits, 3rd ed, International Atomic Energy Agency, Vienna, 2021. https://www-pub.iaea.org/MTCD/Publications/PDF/PUB1923_web.pdf

[2] Dondi M, Torres L, Marengo M, Massardo T, Mishani E, Ellmann AV, Solanki K, Delaloye AB, Lobato EE, Miller RN, Paez D, Pascual T. Comprehensive auditing in nuclear medicine through the international atomic energy agency quality management audits in nuclear medicine (QUANUM) program. Part 1: The QUANUM Program and Methodology. Semin Nucl Med. 2017; 10.1053/j.semnuclmed.2017.07.003. 10.1053/j.semnuclmed.2017.07.003 28969766

[3] Dondi M, Torres L, Marengo M, Massardo T, Mishani E, Ellmann AV, Solanki K, Delaloye AB, Lobato EE, Miller RN, Paez D, Pascual T. Comprehensive auditing in nuclear medicine through the international atomic energy agency quality management audits in nuclear medicine program. Part 2: Analysis of Results. Semin Nucl Med. 2017; 10.1053/j.semnuclmed.2017.07.004. 10.1053/j.semnuclmed.2017.07.004 28969767

[4] Australian Commission on Safety and Quality in Health Care. National Safety and Quality Health Service Standards, 2nd edition, version 2, Sydney: ACSQHC; 2021. http://www.safetyandquality.gov.au

[5] World Health Organisation. Your 5 moments for hand hygiene. 2009. https://cdn.who.int/media/docs/default-source/integrated-health-services-(ihs)/infection-prevention-and-control/your-5-moments-for-hand-hygiene-poster.pdf?sfvrsn=83e2fb0e_21. Accessed Jul and Aug 2023

[6] European Commission. Radiation Protection No. 198. QuADRANT – a European study on clinical audit of medical radiological procedures. Current status and recommendations for improving uptake and implementation of clinical audit of medical radiological procedures. Luxembourg: Publications office of the European Union. 2022; 10.2833/468186.

[7] Delgado Bolton RC, Giammarile F, Howlett DC, Jornet N, Brady AP, Cofey M, Hierath M, Clark J, Wasak W (2023). The QuADRANT study: current status and recommendations for improving uptake and implementation of clinical audit of medical radiological procedures in Europe—the nuclear medicine perspective. Eur J Nucl Med Mol Imaging.

[8] Berman J, Moadel RM, Goldman-Yassen AE, Abraham T, Ye K, Volansky J, Goldberg-Stein S (2020). Impact of patient-centered care on the patient experience in nuclear medicine. Curr Probl Diagn Radiol.

[9] Moncayo VM, Applegate KE, Duszak R, Barron BJ, Fitz J, Halkar RK, Lee DJ, Schuster DM (2015). The nulear medicine therapy care coordination service: a model for radiologist-driven patient-cenered care. Acad Radiol.

[10] International Atomic Energy Agency. QUANUM: patient Journey and other surveys. https://www.iaea.org/resources/hhc/nuclear-medicine/quanum/patient-journey. Accessed Sep and Oct 2023

